# QDECR: A Flexible, Extensible Vertex-Wise Analysis Framework in R

**DOI:** 10.3389/fninf.2021.561689

**Published:** 2021-04-22

**Authors:** Sander Lamballais, Ryan L. Muetzel

**Affiliations:** ^1^Department of Clinical Genetics, Erasmus MC University Medical Center, Rotterdam, Netherlands; ^2^Department of Epidemiology, Erasmus MC University Medical Center, Rotterdam, Netherlands; ^3^Department of Child and Adolescent Psychiatry/Psychology, Erasmus MC University Medical Center, Rotterdam, Netherlands

**Keywords:** cerebral cortex, neuroimaging, statistics, surface-based, vertex-wise analysis

## Abstract

The cerebral cortex is fundamental to the functioning of the mind and body. *In vivo* cortical morphology can be studied through magnetic resonance imaging in several ways, including reconstructing surface-based models of the cortex. However, existing software for surface-based statistical analyses cannot accommodate “big data” or commonly used statistical methods such as the imputation of missing data, extensive bias correction, and non-linear modeling. To address these shortcomings, we developed the QDECR package, a flexible and extensible R package for group-level statistical analysis of cortical morphology. QDECR was written with large population-based epidemiological studies in mind and was designed to fully utilize the extensive modeling options in R. QDECR currently supports vertex-wise linear regression. Design matrix generation can be done through simple, familiar R formula specification, and includes user-friendly extensions for R options such as polynomials, splines, interactions and other terms. QDECR can handle unimputed and imputed datasets with thousands of participants. QDECR has a modular design, and new statistical models can be implemented which utilize several aspects from other generic modules which comprise QDECR. In summary, QDECR provides a framework for vertex-wise surface-based analyses that enables flexible statistical modeling and features commonly used in population-based and clinical studies, which have until now been largely absent from neuroimaging research.

## Introduction

The cerebral cortex is integral to human psychological and physical functioning, and has been studied for centuries (Fan and Markram, 2019). Modern neuroimaging techniques have enabled non-invasive assessment of the cortex, which has led to a myriad of studies elucidating the antecedents and consequences of typical and atypical cortical features. One common method for obtaining *in vivo* brain scans is with magnetic resonance imaging (MRI). The images begin as a grid of 3-dimensional grayscale pixels (voxels) which are commonly further processed by classifying (segmenting) the voxels into gray matter, white matter and cerebrospinal fluid components (Wright et al., 1995). Other tools subsequently trace and isolate the cortex to create surface-based representations of the brain as a series of interconnected points (vertices) forming a 2D mesh (Dale and Sereno, 1993; Fischl, 2012). Specific characteristics of the cortex like its thickness or curvature can be derived from these surface representations at unique locations across the cortical mantle. These maps tend to consist of hundreds of thousands of vertices, allowing the study of the cerebral cortex at a fine-grain resolution. With advent of *population neuroscience* (Paus, 2010) and the introduction of several large-scale open-access neuroimaging initiatives (Alfaro-Almagro et al., 2018; Casey et al., 2018), it is imperative that the neuroimaging community has a repertoire of tools available which are able to accommodate these massive, high dimensional data sets.

Tools for vertex-wise analyses of brain imaging data have been around since the creation of surface-based cortical models. One widely used tool which implements the linear model is Qdec^1^, which stands for Query, Design, Estimate, Contrast. Qdec is bundled with FreeSurfer (Fischl, 2012), an open source software suite designed to generate surface-based maps of the brain from structural MRI data. Qdec facilitates whole-brain, vertex-wise analyses from a graphical user interface. Though brilliantly user-friendly, the interface has limitations including model specification (e.g., restrictions on the number of continuous and categorical variables that can be used) and handling of missing data. Qdec is the front-end interface which was built on top of the mri_glmfit program; a tool which was written in C++, works from the command line, and can handle larger datasets and more complicated design matrices. Another tool, SurfStat (Worsley et al., 2009), was developed in MATLAB and has a number of user-friendly features that mri_glmfit does not have, including formula-based creation of design matrices. SurfStat is still widely used (e.g., Albaugh et al., 2019), but has not been updated since 2008 and requires a MATLAB license.

The field of neuroimaging is rapidly developing, particularly with studies generally growing in sample size due to the advent of open databases, consortia collaborations, and population neuroscience initiatives (Paus, 2010). These studies, increasingly more epidemiologic in nature, require analytical tools that can handle statistical and epidemiological characteristics like big datasets, correction for confounding and selection bias, allowing imputed data to account for missingness, and creating more flexibility in statistical model specification (Smith and Nichols, 2018). The previously described vertex-wise analysis tools were designed with vertex-wise analyses as their core purpose, but may lack features that are now crucial to begin integrating into neuroimaging as common practice to ensure proper analysis and interpretation of the data. Furthermore, expansion of features of those tools is not always straightforward as they were not designed in a modular fashion. Lastly, each tool is designed within a software framework which was not originally designed with statistical computing in mind.

We designed the QDECR package, a flexible and extensible R package for vertex-wise analyses. R is a programming language designed around statistical computing (R Core Team, 2016) and has become increasingly popular in academia and neuroimaging (Muschelli et al., 2019). More importantly, R has a standardized syntax for statistical modeling, arguably has the most extensive statistical functionality of all existing programming languages, and its codebase is improving and expanding every day through a large user base and open source framework. We designed QDECR to fully utilize R’s existing statistical infrastructure. We also designed QDECR to use the same user-friendly syntax as all other R modeling functions, so new users are immediately familiar with the QDECR syntax. Finally, we designed it to be an extensible and modular framework, where advanced users can implement their own type of statistical analyses on a vertex-wise level while still using core features of the framework (e.g., reading data, generating figures). In this manuscript we will describe the structure and features of the QDECR package.

## Materials and Methods

### General Work Flow

**FIGURE 1:**
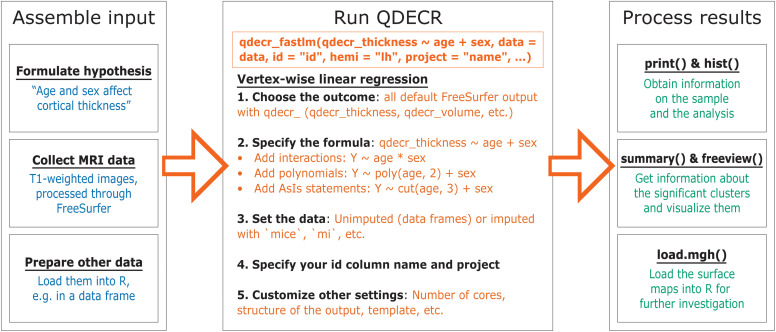
General workflow of the QDECR package, from data collection to post-processing.

The general work flow of the QDECR package is shown in Figure 1.

#### Input: FreeSurfer-Processed Images

Two sources of data need to be available in order to conduct analyses. The first stream of data consists of the T_1_-weighted MR images, which have been fully processed through the FreeSurfer analysis suite (i.e., “recon-all”). Of note, after running the full primary “recon-all” reconstruction, users also need to run the “qcache” processing with “recon-all.” Activating the “-qcache” flag will co-register a given dataset into a standard coordinate system and spatially smooth the surface-based maps with a set of full width half max (FWHM) values. It outputs these maps in.mgh file format (Massachusetts General Hospital) in the “surf” subdirectory of the FreeSurfer output. As part of the FreeSurfer installation, the “SUBJECTS_DIR” environmental variable is set to indicate the directory where all of the subject data are stored; QDECR will recognize this environmental variable, and users can set (or override) it via an optional argument when calling QDECR.

#### Input: Phenotype/Covariate Data

The second stream of data involves the other information relevant to the research question, specifically the phenotypic information of interest and covariates (e.g., age and sex). These data should be loaded into R with the user’s method of choice, and ideally stored as a standard (imputed) data frame object. Furthermore, the phenotype data must include the identifier which was used to store the MRI data in order to link the two data types during analysis (i.e., the identifier variable the MRI data are stored on).

#### Analysis With QDECR

The next step is to run one of the analysis functions from the QDECR package, for example “qdecr_fastlm” for linear regression. At minimum, the following input arguments need to be specified:

• “formula”: a formula object, specifying the linear model to be used;
• “data”: a data frame containing the non-vertex (e.g., phenotype/covariate) data related to the research question;
• “id”: the name of the column in the data frame that identifies each subject;
• “hemi”: a hemisphere (“lh” for left, “rh” for right);
• “project”: a project name used for labeling output files.

As an example, to run a vertex-wise analysis to study the effect of age and sex on cortical thickness of the left hemisphere, the R code would be:

“qdecr_fastlm(qdecr_thickness ∼ age + sex, data = pheno, id = “id,” hemi = “lh,” project = “test”)”

where “pheno” is an R data frame object containing at least the columns “id”, “age,” and “sex”. All rows in “pheno” must correspond to an existing MRI session in “SUBJECTS_DIR”. During the analysis, information on the input data as well as the progress of the analysis will be printed on the console. The analysis will generate a number of files on disk (Table 1). The output of the analysis can be stored directly into an R variable, or it can be loaded back in at a later point in time.

**TABLE 1:** Overview of the output files.

**Name**	**Per stack**	**Description**
“project”.rds	no	A file that stores the qdecr output object and can be reloaded with “qdecr_load.”
finalMask.mgh	no	The final mask that was used for the analyses.
fwhm.dat	no	A file containing the estimated smoothness.
significant_clusters.txt	no	Contains all significant clusters (the output of “summary(vw, annot = TRUE)”).
stack_names.txt	no	Contains the link for variable name-stack number.
stack*.coef.mgh	yes	Contains the vertex-wise regression coefficients from the linear regression.
stack*.se.mgh	yes	Vertex-wise standard errors from the linear regression.
stack*.t.mgh	yes	Vertex-wise *t*-values from the linear regression.
stack*.p.mgh	yes	Vertex-wise *p*-values from the linear regression.
stack*.cache.th*.abs.sig.cluster.mgh	yes	Vertex-wise log10-transformed *p*-values of the cluster-wise significance.
stack*.cache.th*.abs.sig.cluster.summary	yes	Text file with summary information about clusters from the “mri_surfcluster” call.
stack*.cache.th*.abs.sig.masked.mgh	yes	Vertex-wise values after setting the non-cluster vertices to zero.
stack*.cache.th*.abs.sig.ocn.annot	yes	Vertex-wise annotations for the clusters to which each vertex belongs.
stack*.cache.th*.abs.sig.ocn.mgh	yes	Vertex-wise values for the clusters to which each vertex belongs.
stack*.cache.th*.abs.sig.voxel.mgh	yes	Vertex-wise value for the corrected voxel-wise significance.

The “project”.rds file will have the name of the outputted project name. The “stack*” names will be replaced with the stack number, e.g., “stack1.” The “th*” names will be replaced with the threshold of the cluster-wise threshold. All “.cache.” files are output from mri_surfcluster.

In the current version of QDECR, results which are corrected for multiple comparisons (i.e., tests across all vertices) are by default also saved. This is done automatically in “qdecr_fastlm” using pre-cached smoothed Gaussian Monte Carlo – known as MCZ – simulations on a cluster level (Hagler et al., 2006). The cluster-forming threshold can be changed by specifying the “mcz_thr” argument [default = 0.001 based on previous work showing correspondence with full permutation tests (Greve and Fischl, 2018)]. The cluster-wise *p*-values are further corrected for performing additional analyses (e.g., in both hemispheres), which can be set with the “cwp_thr” argument (default = 0.025, which is 0.05 Bonferroni corrected for running both left and right hemispheres).

The “qdecr_fastlm” function has many, additional arguments that users can specify. Information on the function and its arguments can be obtained by calling “?qdecr_fastlm.” Several arguments may be of particular interest to users. First, users can a path to SUBJECTS_DIR into “qdecr_fastlm” directly with the “dir_subj” argument, and to FREESURFER_HOME with the “freesurfer_home” argument. Second, the “target” argument allows for specification of the target template to use. Users can input templates that are available in SUBJECTS_DIR, but note that “–qcache” must be run with whichever target template has been specified. By default, the “fsaverage” template is used. Third, users can differentiate which level of smoothing (i.e., FWHM) should be used by using the “fwhm” argument, which is set to 10 by default.

#### Inspection of QDECR Output

The output of the analysis can be explored with an array of functions within the QDECR package. Most of these functions were built on top of commonly used R functions. For example, the “print” and “summary” functions – which are familiar to most R users – can be used to extract information about the analysis and the significant brain areas (clusters) identified, respectively. Furthermore, QDECR provides functions to visualize the data (e.g., “hist” and “freeview”).

### Internal Structure

The field of neuroimaging has grown extensively over the last few decades, and a wealth of analysis methods have been developed. This can be daunting for new users, who would benefit from user-friendly and restricted analysis software. However, such restrictions may deter advanced users who require flexibility in applying their methods. QDECR was designed with both audiences in mind: straightforward and intuitive to use for beginners, yet flexible and extensible for advanced users. To achieve this, QDECR was designed to contain six modules:

1. Input checking. All input arguments undergo integrity checks. For example, provided paths are checked if they already exist in the system, and datasets are checked for the presence of the variables in the model specification, i.e., the formula.
2. Model preparation. The first steps of the statistical modeling are done here. The user-specified model is created and all steps that can be done before the vertex-wise calculations are processed. For example, in linear regression the portion of the design matrix that is common to all vertices is generated here.
3. Loading the vertex-wise data. In this step, the dataset and the provided paths are used to load in the vertex-wise data into a file-backed matrix.
4. Vertex-wise analysis. This module builds upon step 2 and runs the statistical model for every vertex. The output of each model is stored in dedicated file-backed matrices.
5. Multiple testing correction. Once all analyses are done, multiple testing correction can be applied across all vertices.
6. Output generation. An R object is compiled to contain all the information on the QDECR call, and output files are generated on disk to store the results more permanently.

These modules are implemented into the “qdecr” function. At its core, “qdecr” can handle any statistical model that is entered as long as a model preparation module (module #2) and a corresponding vertex-wise analysis module (module #4) exist. Functions like “qdecr_fastlm” are wrappers that automatically use the appropriate modules in “qdecr” to perform vertex-wise linear regression. Thus, users who only want to perform analyses do not have to think about any of the modules nor the underlying “qdecr” function, while advanced users can use the framework to more easily implement new types of models.

### Formula Objects

An important part of regression modeling is the creation of a design matrix. R uses formula objects in building design matrices. Formulas usually have three components: (Fan and Markram, 2019) the left hand side (the outcome or dependent variable) (Wright et al., 1995) the right hand side (the determinants or independent variables) and (Dale and Sereno, 1993) a tilde to separate the sides. For example, in the formula “qdecr_thickness ∼ age + sex,” the qdecr_thickness is clearly defined as the outcome variable, and age and sex are denoted as the determinant variables. Using R formula objects for design matrix creation has a number of strengths:

• Categorical variables (like “sex”) are automatically recoded. By default, the levels will be dummy-coded according to the default behavior of linear regression in R, but other contrasts are available.
• Interaction terms can be introduced using the “^∗^” (main effects plus interaction) or “:” (interaction only) symbols, for example “qdecr_thickness ∼ age ^∗^ sex.”
• Variables can be customized within the formula, for example by adding polynomial terms (e.g., “poly(age, 3)”), adding splines (e.g., “splines::ns(age, 3)”), standardizing a variable (e.g., “scale(age)”) and recoding of variables (e.g., “cut(age, 3)”).
• New features for formulas can be seamlessly introduced, such has been done with the “Formula” package (Zeileis and Croissant, 2010).

Thus, R formula objects – when used properly – allow for intuitive and extremely powerful behavior related to the creation of a design matrix. QDECR builds upon these principles, and in general most functions that manipulate formula objects will automatically work in QDECR as well, offering users continuity in syntax they already know from R.

Note that QDECR can handle all vertex-wise measures that FreeSurfer outputs by default, and the names are simply the FreeSurfer-assigned names preceded by “qdecr_” (Table 2), such as “qdecr_thickness” and “qdecr_area.pial.” The only modification is “w-g.pct,” which is written as “qdecr_w_g.pct” as the hyphen (or minus sign) has a specific meaning in R formula objects. In certain cases, users may choose to create custom surface maps (e.g., functional activation maps). QDECR can be used to analyze those maps, by specifying the “custom_measure” argument of “qdecr_fastlm.” Users should supply the name of the vertex measure preceded by “qdecr_” (e.g., “qdecr_radialdistance”). Furthermore, the surface files must be placed in the “surf” directory of the FreeSurfer output of each participant. Finally, the surface files have to follow the same naming convention as the other surface maps (e.g., “lh.radialdistance.fwhm10.fsaverage.mgh”).

**TABLE 2:** Overview of the surface-based measures.

**Surface measure in FreeSurfer**	**Name in QDECR**	**Description**
area	qdecr_area	Surface area of the white matter surface
area.pial	qdecr_area.pial	Surface area of the pial matter surface
curv	qdecr_curv	Smoothed mean curvature
jacobian_white	qdecr_jacobian_white	The Jacobian of the spherical transformation
sulc	qdecr_sulc	Average convexity compared to the average surface
thickness	qdecr_thickness	Cortical thickness; the distance between the white and pial surfaces
volume	qdecr_volume	Cortical volume
w-g.pct	qdecr_w_g.pct	Gray to white signal intensity ratio
white.H	qdecr_white.H	Mean curvature of the white surface
white.K	qdecr_white.K	Gaussian curvature of the white surface

### Statistical Modeling of Linear Regression

The base model implemented in QDECR is a vertex-wise linear regression model with the vertex-wise metric, e.g., cortical thickness, as the outcome. At each vertex, a least squares regression would be performed:

$$\beta ={\left({\text{X}}^{T}\,\text{X}\right)}^{-1}\,{\text{X}}^{T}\,\text{y},$$

where **X** is an *N* (subjects) × *p* (variables) matrix that is the design matrix, y is an *N* × 1 vector with the values at a given vertex, and β is a *p* × 1 vector of regression coefficients.

Running a linear regression for each separate vertex using the default “lm” function from the “stats” package would take a significant amount of time in R as R is an interpreted programming language. In interpreted languages the interpreting and execution of a line of code requires some operation time. Given the thousands of vertices that maps exist of, and given that linear regressions take milliseconds to perform, the compute time can become hours to days. The regression coefficients for all *m* vertices can be determined in a single step with the formula:

$$\text{B}={\left({\text{X}}^{T}\,\text{X}\right)}^{-1}\,{\text{X}}^{T}\,\text{Y},$$

Where **Y** is an *n* × *m* (number of vertices) matrix with all vertex-wise values, and **B** is a *p* × *m* matrix of regression coefficients. Note that the vertex measures are the outcome, and thus the design matrix for all vertices is identical. In order to decrease run time QDECR therefore only builds the design matrix once.

The matrices may become exceedingly large given thousands of vertices, thousands of participants and tens of imputed datasets. This would then exceed the RAM size of the RAM size of consumer grade computers. To avoid this, QDECR internally splits **Y** into “chunks,” or smaller partitions, so that the analyses can be run in smaller sets limiting the amount of required RAM at a given moment. By default, “qdecr_fastlm” creates chunks of 1,000 vertices, but the size can be fine-tuned to a given setup (e.g., number of subjects, RAM availability, imputed datasets, etc.) with the “chunk_size” argument.

### Handling Imputed Data

Missing covariate or phenotypic data in datasets can pose problems for statistical analyses. Previous vertex-wise tools require complete data, and thus any subjects with missing covariate or phenotypic data would have to be excluded for analysis, which could lead to loss of power and an increase in bias (Rubin, 1976). Rather than using only complete observations, methods have been developed to impute the missing data, typically under the assumption that the missingness is random and that the missingness can be predicted from other available data. To account for uncertainty in the imputation process, the imputation is repeated to generate several imputed datasets. For users who decide imputations are useful and feasible for their particular set of analyses, QDECR was designed to handle such imputed datasets from the most commonly used R imputation packages. Internally, QDECR uses a function called “imp2list” that converts any prespecified data object to a list of data frames. Consequently, “qdecr_fastlm” accepts the following object types for its “data” argument: Data frames, matrices, and lists of data frames, as well as imputed objects from the following R packages: mice (van Buuren and Groothuis-Oudshoorn, 2011), mi (Su et al., 2011), amelia (Honaker et al., 2011), and missForest (Stekhoven and Buhlmann, 2012). Furthermore, users can implement methods for new classes by converting their object to a list of datasets. In regression analyses, the estimates across the imputed datasets are pooled using Rubin’s rules (Rubin, 1987).

### Proof of Principle for Vertex-Wise Linear Regression

To illustrate the QDECR package we performed vertex-wise analyses in 1,000 randomly selected participants from the UK Biobank (Alfaro-Almagro et al., 2018). The participants had a mean age of 63.9 years (standard deviation: 7.7, range: 47.1 – 80.0) and 52.8% was female. The T_1_-weighted images were processed with FreeSurfer version 6.0 (Fischl, 2012). Additionally, in order to facilitate reproducible benchmarking and testing, a set of 10,000 simulated surface-based cortical thickness maps have also been made publicly available alongside a full installation of QDECR at Code Ocean (Lamballais and Muetzel, 2020), and can be freely explored and tested via the web interface^2^. A full tutorial on how to use QDECR can be found in the Supplementary Materials (Section 1) and via the GitHub repository^3^.

## Results

### Age Associations: Example in the UK Biobank

Within the UK Biobank sample, we aimed to study the association between age and vertex-wise cortical thickness, adjusted for sex. This can be achieved by running the following code in R:

vw <- qdecr_fastlm(qdecr_thickness ∼ age + sex, data = pheno, id = “id,” hemi = “lh,” project = “test_project”)

The formula (i.e., qdecr_thickness ∼ age + sex) captures cortical thickness as the dependent variable, and age and sex as the independent variables. The variable “pheno” contains the information on age and sex per participant, and the identifier is “id.” By specifying “hemi = “lh””, we specify that the left hemisphere should be analyzed. Finally, the project name “test_project” is used, which will be incorporated in the names of the files that will be written to disk. The output is stored in the variable “vw.”

**FIGURE 2:**
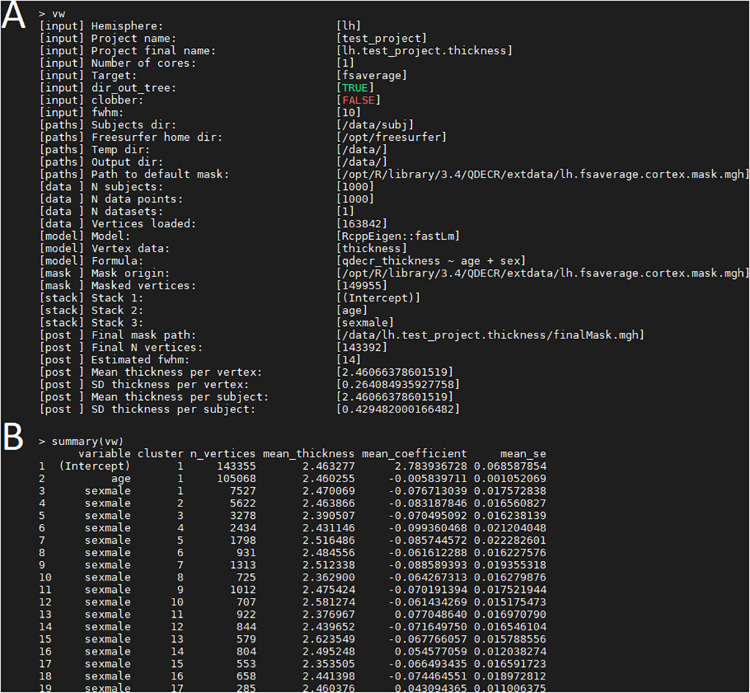
Examination of the QDECR analysis on the association of age and cortical thickness in a subset of the UK Biobank. **(A)** The “print” function returns the key information of the analyzed project, including the input arguments, the included formula, the size of the dataset and the number of included vertices. **(B)** An example of output from the “summary” function. Each row represents a statistically significant cluster.

**FIGURE 3:**
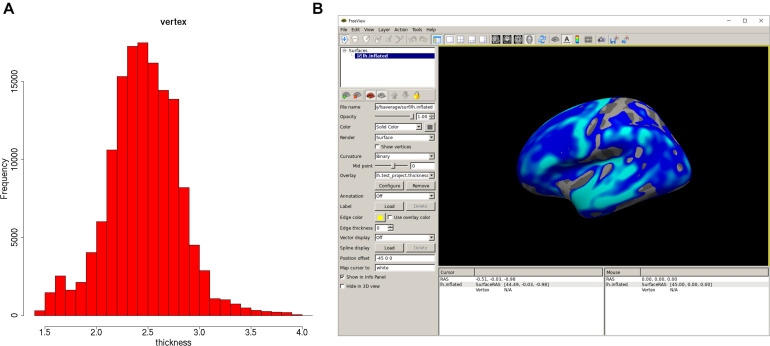
Plots of the QDECR analysis results. **(A)** A histogram of the vertex-wise mean cortical thickness for the study sample, generated with “hist.” **(B)** FreeView can be called with the “freeview” function. This panel displays the vertex-wise map for the effect of age on cortical thickness.

Once the analysis is done, a summary of the analysis can be viewed with “print(vw)” (Figure 2A). The significant clusters can be tabulated with “summary(vw)” (Figure 2B), which shows that a number of clusters have a significant association with age. Further inspection of the vertex-wise data can be done with “hist(vw)” (Figure 3A), which generates a histogram of the vertex-wise mean cortical thickness. The results can be visualized with the FreeSurfer FreeView tool by typing “freeview(vw)” (Figure 3B). Within the FreeView visualization it is clear that cortical thickness generally decreases with age, particularly in the temporal lobe and the precentral gyrus.

### Age Associations: Comparison to mri_glmfit

In order to demonstrate the accuracy and consistency of the tool, we compared the results from the previous paragraph to those of mri_glmfit using the same data from the UKBB. A linear model for the association of age and sex with cortical thickness was generated in mri_glmfit with a DOSS (Different Offset, Same Slope) design. A full description of the input and code is given in the Supplementary Materials (Section 2). The vertex-wise regression coefficients – or betas – for age were examined. The mean absolute difference in the regression coefficients for age across the tested vertices was 7.9 ⋅ 10^–7^, which arose from differences in rounding. The correlation between the betas was near perfect (Pearson’s *r* = 1.00, Spearman’s ρ = 0.9999954).

### Performance Benchmark

**FIGURE 4:**
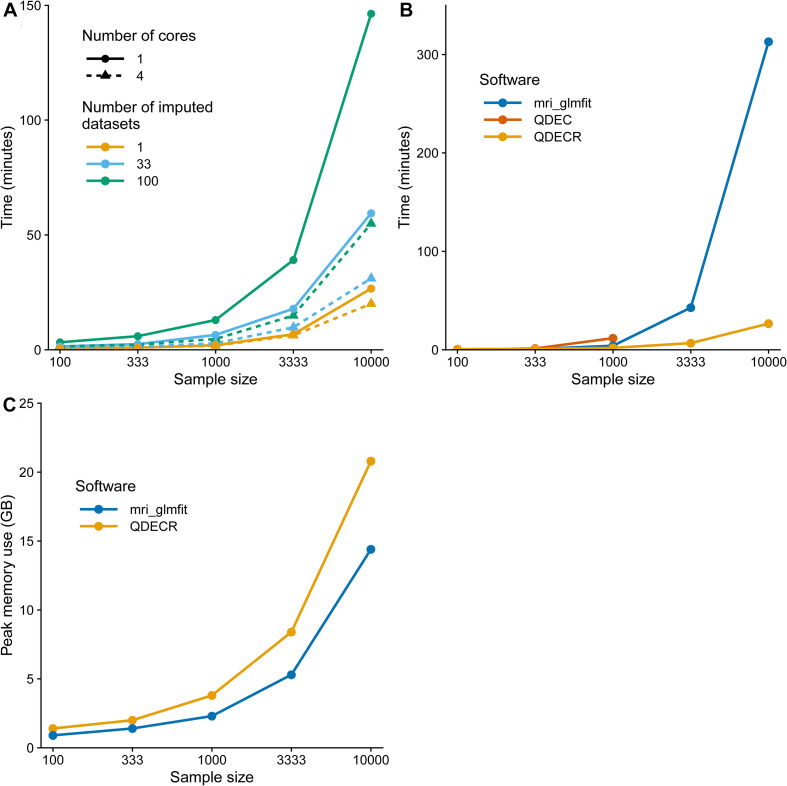
Computation time benchmark of the QDECR package. **(A)** Displays the computation time of an analysis with QDECR when varying sample size, number of cores (threads) used and number of imputed datasets that were included. **(B)** Compares the computation time for mri_glmfit, QDEC and QDECR when using a single core (thread) and unimputed data. For QDEC we used the time that the analysis took, without loading or assembling of the data or the multiple testing correction. For mri_glmfit we measured the time it took to run three commands: (Fan and Markram, 2019) mri_preproc to assemble the FreeSurfer output, (Wright et al., 1995) mri_glmfit to run the analysis and (Dale and Sereno, 1993) mri_glmfit-sim to perform the multiple testing correction. **Note: QDEC returned errors when attempting to run the analyses on 3,333 and 10,000 participants and is thus not represented for those sample sizes in panel **(B)**. **(C)** Compares the peak memory use in gigabytes (GB) for mri_glmfit and QDECR when using a single core (thread) and unimputed data.

In order to demonstrate how QDECR performs in terms of compute time in comparison to other tools, we used simulated data to benchmark the performance of “qdecr_fastlm.” We studied the influence of sex and age on cortical thickness of the left hemisphere in 100, 500, 1,000, 5,000 and 10,000 participants. Further, we tested the impact of multiple imputation by generating 1, 33 or 100 imputed datasets. Lastly, we studied the impact of parallel processing by using 1 or 4 CPU cores. Figure 4A contains the results of the benchmark. The time it took to perform an analysis on unimputed data ranged from 0.8 min for a sample of 100 datasets to 26.7 min for a sample of 10,000 datasets. When the dataset with 10,000 was further imputed 100 times for missing covariates, the time increased to 146.4 min (i.e., 448% longer than unimputed). The performance can be boosted by recruiting more CPU cores for the analysis. For example, using 4 cores compared to 1 core on 10,000 datasets with 100 imputed datasets dropped the analysis time from 146.4 to 54.9 min (i.e., 62.5% reduction).

We further compared the QDECR performance with Qdec and mri_glmfit (Figure 4B). Both Qdec and mri_glmfit were faster than QDECR on samples of 100 individuals, and slower for samples with 333 participants and more. Furthermore, Qdec was not able to finish the analyses on 3,333 and 10,000 participants due to errors that arose when merging the underlying MRI data into a single set. While mri_glmfit did succeed in running the analyses, compared to QDECR it was much slower for the set of 3,333 individuals (42.8 vs. 6.8 min with QDECR) and 10,000 individuals (313.1 vs. 26.7 min with QDECR). Finally, we compared the peak memory use of QDECR with that of mri_glmfit (Figure 4C). Overall, QDECR had a higher peak memory use than mri_glmfit. Where QDECR reached a peak memory use of 8.4 GB for 3,333 individuals and 20.8 GB for 10,000 individuals, mri_glmfit used 5.3 and 14.4 GB, respectively.

## Discussion

QDECR provides a framework to perform vertex-wise analyses in R. It has the same base functionality as other vertex-wise tools and adds several functionalities. We have shown that QDECR runs faster on large datasets than other tools, and can additionally handle imputed datasets to minimize bias or loss of power due to exclusion of participants. Moreover, we aimed to maximize user friendliness for individuals familiar with R through the implementation of formula objects to handle design matrix specification and through writing functions with similar arguments as other base functions. Finally, QDECR sets the stage for further development of statistical applications to study the cerebral cortex in population neuroscience settings.

QDECR has a number of limitations. The primary focus of the package has been to implement vertex-wise analyses in R. In contrast, glmfit has a myriad of options for the MRI data available. It works with both voxel-wise and vertex-wise data with all volume files that are recognized by the FreeSurfer mri_convert function (.mgh,.nii, etc.). It also has several options related to the analysis that are not available in QDECR yet, such as different methods for multiple testing correction (e.g., permutation testing) and weighted least squares. However, QDECR is still in development, and these options will likely be available in the future. Another limitation for part of the potential users is that QDECR is only available in R. Qdec will therefore remain more feasible for users with little programming experience, and MATLAB and Python users would have to learn basic R skills in order to use it. Furthermore, developing new modules requires mastery of R. Still, we opted for R as it provides an ideal environment to further develop the statistical options for vertex-wise analyses. Furthermore, R is gaining popularity in medical research, especially with the advent of Bioconductor (Huber et al., 2015) and more recently Neuroconductor (Muschelli et al., 2019).

While QDECR presents a substantial contribution to vertex-wise analyses, several areas of opportunity for expansion and improvement exist. First, at the moment only the general linear model is implemented. We envision logistic regression, linear mixed models, and structural equation models to be the next key targets for future implementation. Next, though QDECR relies on multiple testing correction that is native to the FreeSurfer library, new modeling techniques may require new methods for adjusting analyses for multiple comparisons. Thus, another target for development is implementing permutation testing and other state-of-the-art methods in the field of neuroimaging. Finally, QDECR in its first implementation can handle the mgh file format and assumes a FreeSurfer image reconstruction. In the future, new methods should be implemented to accommodate other file format types [e.g., Minc/Civet (Ad-Dab’bagh et al., 2006)], and allow for 3D voxel data in addition to surface data (e.g., Nifti format data).

## Conclusion

QDECR extends the capabilities of existing whole-brain vertex-wise statistical software for neuroimaging data analysis, allowing for larger (population-based) datasets, incorporation of novel epidemiological and statistical concepts, and elegant expansion within the widely used and open-source R statistical framework.

## Software Availability Statement

The code is made available at https://github.com/slamballais/QDECR. Supporting information and further tutorials can be found at https://www.qdecr.com. QDECR uses the GNU General Public License version 3.0 (GPL-3).

## Data Availability Statement

The data analyzed in this study is subject to the following licenses/restrictions: legal and informed consent restrictions. Requests to access these datasets should be directed to the UK Biobank (http://www.ukbiobank.ac.uk).

## Ethics Statement

The studies involving human participants were reviewed and approved by the NHS National Research Ethics Service (ref 11/NW/0382). The patients/participants provided their written informed consent to participate in this study.

## Author Contributions

RM conceived the package. SL and RM designed the package. SL adapted all the functions and code, and optimized the performance. Both authors wrote the manuscript and approved the final manuscript.

## Conflict of Interest

The authors declare that the research was conducted in the absence of any commercial or financial relationships that could be construed as a potential conflict of interest.

## Footnotes

1 https://surfer.nmr.mgh.harvard.edu/

2 https://codeocean.com/capsule/6804031/tree/v2

3 https://github.com/slamballais/QDECR
