# Supplementary material for: QDECR: A Flexible, Extensible Vertex-Wise Analysis Framework in R
Source: Front Neuroinform. 2021 Apr 22;15:561689. doi: 10.3389/fninf.2021.561689 (PMC8100226; doi:10.3389/fninf.2021.561689)
Supplement: Supplementary file 1 [file Data_Sheet_1.docx]

**Supplementary Materials**

**1. Tutorial on using QDECR**

*1.1 Installation of QDECR*

R version 3.4.3 or higher is required for QDECR. The QDECR package can be downloaded and installed from GitHub using the `install_github` function from the `devtools` package. QDECR has several R package dependencies which will automatically be installed via this method.

| # install (only required the first time) and load QDECR  if (!requireNamespace("devtools")) install.packages("devtools")  if (!requireNamespace("QDECR")) devtools::install_github("slamballais/QDECR")  library(QDECR) |
| --- |

*1.2 Setting up the programming environment*

The FreeSurfer analysis suite needs to be configured according the default FreeSurfer documentation, including setting (and exporting) the `FREESURFER_HOME` variable in Unix and running a setup script (see <https://surfer.nmr.mgh.harvard.edu> for details on installing and configuring FreeSurfer). Next, the other data relevant to the research question (i.e., phenotype data) need to be loaded into R. This can be done in any way that R supports. For example, below a .csv file is read in as a data frame and saved into the variable `data`. Imputed datasets can be used as phenotype input to QDECR without any additional settings, as long as the data are stored in an accepted format (e.g. a `mids` object from the `mice` package or a list of data frames). No special steps need to be undertaken to differentiate between unimputed and imputed datasets, and the sole requirement is that an `id` variable exists which can be mapped to the processed MRI data in the `SUBJECTS_DIR`.

| # Run within the (BASH) Unix environment  export FREESURFER_HOME=/opt/freesurfer/6.0.0  export SUBJECTS_DIR=/data/subj  source ${FREESURFER_HOME}/SetupFreeSurfer.sh |
| --- |

*1.3 Running an analysis*

Once all data are prepared and the proper environmental variables are set, a QDECR analysis function can be called. The `qdecr_fastlm` function performs linear regression at every vertex, one hemisphere at a time. At minimum, the function requires input of [1] a formula, [2] a phenotype dataset, e.g. a data frame, [3] the name of the identifier column in the dataset, [4] the hemisphere ("lh" or "rh") and [5] a name for the project that will be assigned to any output files. Formulas are constructed in an identical fashion to other formulas in R. For example, the formula `qdecr_thickness ~ age + sex` creates a model where cortical thickness is the outcome (dependent variable) and age and sex are the predictors (independent variables). All options for the vertex measures are displayed in Table 2.

| # load in phenotypic data, e.g. from a .csv file  pheno <- read.csv("pheno.csv")  # run linear regressions with cortical thickness as outcome, and age and sex as determinants  vw <- qdecr_fastlm(qdecr_thickness ~ age + sex,  data = pheno,  id = "id",  hemi = "lh",  project = "test_project") |
| --- |

The `qdecr_fastlm` function has additional arguments to further customize analyses, which include the following (see `?qdecr_fastlm` in the R command line for all options):

- The target template can be set by specifying the `target` argument (default: "fsaverage"). This must correspond to the template used in the “-qcache” stage of “recon-all”.
- The FWHM smoothing level is set to 10 mm by default (5 mm for `pial_lgi`), but can be changed by setting the `fwhm` argument.
- If the variable `SUBJECTS_DIR` has not been set in the Unix environment, then it can be specified in `qdecr_fastlm` with `dir_subj`.
- QDECR runs on one core by default, but using multiple cores (i.e. parallel computing) can be done by setting the `n_cores` argument.
- During the analysis QDECR writes temporary files to the output directory, which will be deleted at the end of the analysis. The temporary files are used to store the vertex-wise data and vertex-wise results on disk, to reduce random-access memory (RAM) consumption. An alternative directory can be set using the `dir_tmp` argument, and the temporary files will not be deleted if `clean_up_bm` is set to FALSE.

*1.4 Viewing results*

While `qdecr_fastlm` is running it will output information on the console, including all baseline characteristics of the project, progress on loading in the vertex-wise data and progress on the analyses.

Once the `qdecr_fastlm` routine is finished there will be two sources of output: [1] the files on disk (Table 1), and [2] the object returned by `qdecr_fastlm` which we assigned as `vw` in the example above. The `vw` variable can be further manipulated inside R. Information on the analysis can be obtained by typing the variable name on the command line (`vw`), or equivalently by using the print function (`print(vw)`).

To only look at the summary statistics of the statistically significant clusters, i.e. regions in the brain in which the predictor associates with the outcome, the summary function can be used (`summary(vw)`). This will display the significant clusters across all variables, and specifies for each cluster characteristics like the cluster’s size, mean regression coefficient and mean vertex value (e.g. cortical thickness). The `summary` function can be configured to also output annotation information per cluster (`summary(vw, annot = TRUE)`), by default based on the FreeSurfer automatic cortical parcellation.

| # summary information  vw # equivalent to print(vw)  summary(vw)  summary(vw, annot = TRUE) |
| --- |

*1.5 Plotting*

Histograms of the vertex data can be made with the base-R `hist` function. By default, `hist` plots a histogram where each value is the mean value (e.g. mean thickness) across all subjects for a given vertex (Figure 3A). Alternatively, if `qtype = "subject"` is set, `hist` will plot a histogram where each value is the mean value (e.g. mean thickness) for a given person across all vertices. All other available arguments in the `hist` also function within QDECR.

Functions like `qdecr_fastlm` automatically outputs .mgh files that contain surface-based maps of the model statistics. For example, `qdecr_fastlm` outputs maps for the regression coefficients, the standard errors, the t-values and the p-values. These maps are bound to the columns of the design matrix, which can differ from the variables in the supplied formula due to recoding of variables. For example, by default categorical variables (e.g. sex) are dummy-coded, which splits the data over multiple columns or renames the column (e.g. `sexFemale`). Thus, each result from the regression – e.g. coefficients, standard errors, p-values and t-values – will have its own set of maps. Within QDECR, each such estimator is denoted as a "stack". The files that QDECR outputs are identified by the names "stack1", "stack2", etc. The `stacks` function can be used to identify which variables belong to which stacks (e.g., "stack1" is linked to the "(Intercept)" term).

Plotting the maps can be done using the `freeview` function (Figure 3B), which calls the Freeview visualization software from the FreeSurfer suite. First, the stack of interest has to be defined, either with the stack name or the stack number. Second, by default, the regression coefficient map will be plotted, but other maps can be plotted by setting the `type` argument to `se` (standard errors), `t` (t-values) or `p` (p-values). Third, all maps can be thresholded for a given p-value level from the p-value map by setting the `p` argument to a given threshold (default = 0.05). Similarly, "snapshots" (images) the from lateral, medial, superior and inferior views of a hemisphere can be generated with the `qdecr_snap` function. By default, snapshots are stored as ".tiff" files.

| # plotting  hist(vw)  stacks(vw)  freeview(vw, "sexFemale") # equivalent: freeview(vw, 3)  qdecr_snap(vw, "age") # equivalent: qdecr_snap(vw, 2) |
| --- |

*1.6 Loading files and projects*

QDECR vertex-wise functions like `qdecr_fastlm` do not only generate an R output object, but also files on disk. Two groups of files are generated: [1] general files and [2] stack-specific files. The general files include "significant_clusters.txt", which contains the same output as `summary(vw, annot = TRUE)`, and "stacks.txt", which contains the output of `stacks(vw)`. Furthermore, there is a .rds file that contains the R output object and can be loaded back into R using the `qdecr_load` function. This can be useful if users want to reexamine a project that was generated in a previous R session.

The stack-specific files vary per vertex-wise function. The `qdecr_fastlm` function generates stack-wise .mgh files for the vertex-wise regression coefficients, standard errors, t-values and p-values, as well as several other information files (Table 1). The .mgh files can be loaded into R using the `load.mgh` function for further manipulation within R and the `save.mgh` command can be used to save .mgh files to disk.

**2. Comparison with mri_glmfit**

We aimed to compare the results from QDECR with those from mri_glmfit. This was done by examining the association of age and sex with vertex-wise cortical thickness. In QDECR this can be written in a formula as: `qdecr_thickness ~ age + sex`. Here, the `sex` variable is automatically dummy-coded.

To achieve the same model in mri_glmfit, we specified a DOSS (Different Offset, Same Slope) design. The contrast vector specified was: `0 1 0`, denoting that we wanted to retrieve the surface maps for the `age` variable. The contrast vector was stored in the file `contrast.mtx`. The mri_glmfit analysis was run in several steps:

1. A .fsgd file was generated based on the UK Biobank dataset. Such files contain the phenotypic data as well as their associated metadata.
2. The vertex-wise data was merged with `mris_preproc`:

| mris_preproc \  --fsgd /path/to/UKB.fsgd \  --cache-in thickness.fwhm10.fsaverage \  --target fsaverage \  --hemi lh \  --out lh.mri_glmfit.thickness.10.mgh |
| --- |

1. The vertex-wise analysis was run with `mri_glmfit`:

| mri_glmfit \  --y lh.mri_glmfit.thickness.10.mgh \  --fsgd /path/to/UKB.fsgd doss \  --C contrast.mtx \  --surf fsaverage lh \  --glmdir lh.mri_glmfit |
| --- |

Figure 4 was consequently generated from the beta map file (lh.mri_glmfit/beta.mgh).
